# Allocation concealment appraisal of clinical therapy trials using the extended Composite Quality Score (CQS-2)—An empirically based update

**DOI:** 10.3389/fmed.2023.1176219

**Published:** 2023-06-15

**Authors:** Steffen Mickenautsch, Veerasamy Yengopal

**Affiliations:** ^1^Review Centre for Health Science Research, Johannesburg, South Africa; ^2^Department of Community Dentistry, School of Oral Health Sciences, Faculty of Health Sciences, University of the Witwatersrand, Johannesburg, South Africa; ^3^Faculty of Dentistry, University of the Western Cape, Cape Town, South Africa

**Keywords:** Composite Quality Score, systematic review, trial appraisal, clinical trial, allocation concealment

## Abstract

**Objectives:**

The objective of this study was to revise CQS-2/Criterion II concerning allocation concealment appraisal for prospective, controlled clinical therapy trials.

**Methods:**

Meta-analyses of trials with inadequate allocation concealment were tested for in-between trial heterogeneity (*I*^2^ > 0) due to imbalances in baseline variables. Meta-analyses with positive test results were used as a basis to deduce criteria for adequate allocation concealment. The CQS-2/Criterion II was reformulated in line with the findings.

**Result:**

One suitable meta-analysis was identified. Two forest plots with data from five and four trials with inadequate/unclear allocation concealment were selected for testing. In addition, a total of five trials with adequate allocation concealment were identified. The meta-analysis test results were positive, and keywords for the judgment of adequate allocation concealment were extracted verbatim from the text of the meta-analysis. The extracted keywords indicated “central allocation” as the main criterion for adequate allocation concealment. Criterion II of the CQS-2 was revised accordingly.

**Conclusion:**

Criterion II of the CQS-2 trial appraisal tool was revised. The revised appraisal tool was specified as version CQS-2B.

## 1. Introduction

According to the Cochrane Collaboration, the risk of bias in clinical therapy trials, specifically in randomized controlled trials (RCTs), should be assessed by using its Risk of Bias tool, Version 2 (RoB 2) (1). However, the RoB 2 has been found to be of poor inter-rater reliability (Fleiss' Kappa 0.16; 95% CI: 0.08–0.24); its application has been described as complex and demanding (2). The RoB 2 also necessitates intensive formal training and the conduct of pilot runs before it may be correctly applied. Furthermore, integrated teamwork, including expertise in the subject matter of a systematic review, as well as in clinical epidemiology or trial methodology and statistics, is needed (2). Such apparent complexity, together with the poor inter-rater reliability of RoB 2, stands in contrast to the steadily increasing volume of clinical intervention trials worldwide and the subsequent need for timely, uncomplicated, yet effective, and reliable trial appraisal (3).

Against this background, the Composite Quality Score (CQS) is under development as a trial appraisal tool that seems to be an alternative to the RoB 2, based on its epistemological rigor (4), empirical evidence base (5), high inter-rater reliability [Brennan-Prediger coefficient 1.00; 95% CI: 0.94–1.00 (6) and 0.95, 95% CI: 0.87–1.00 (7)], and its apparent ease of application without prior training (6). Its latest version (CQS-2) includes four criteria related to the random allocation to treatment groups, concealment of such allocation, double-blinding, and sample size minimum. The full criteria of the CQS-2 are presented in Table 1. Application of the CQS-2 includes binary trial report rating per appraisal criterion (Scores: 0 = invalid/falsified, 1 = corroborated), multiplication of all criterion scores to an overall appraisal score, and identification of invalid/falsified trial reports based on a zero overall appraisal score (5).

**Table 1:** CQS-2 appraisal criteria.

Criterion I	“Randomization” for allocation to treatment groups is in some form reported in the text
Criterion II	(i) Keeping the random allocation sequence in a locked computer file; and(ii) Translating the sequence into identical, coded, serially administered containers and/or sealed, opaque envelopes; and(iii) Reassuring that the person who generated the sequence did not administer it are in some form reported in the text
Criterion III	Double-blinding or the blinding of at least two out of the three groups: trial participants, trial personnel, and trial outcome assessors in some form reported in the text
Criterion IV	The sample size of any particular treatment group reported in the trial is not < *N* = 100.

Criterion II of the CQS-2 was developed based on evidence from two meta-epidemiological studies (8, 9) to appraise allocation concealment in trials (5). The evidence indicated a statistically significant larger effect estimate for trials with “inadequate” or “unclear” allocation concealment (dSMD 0.15; 95% CI: 0.03–0.28; *I*^2^ = 0%) compared to trials where allocation concealment was judged to be “adequate'. The evidence from both studies combined the results of 379 clinical, dental, oral, and craniofacial trials (5). Adequate allocation concealment was specified as concealment of the random allocation sequence that included (verbatim) the following: “centralized or pharmacy-controlled randomization; coded identical containers administered serially; onsite computer system combined with allocations kept in a locked unreadable computer file; sequentially numbered, sealed, opaque envelopes, and similar schemes ensuring that patient and clinician were unaware of the allocation, along with the reassurance that the person who generated the allocation scheme did not administer it” (9). The CQS-2/Criterion II was formulated accordingly (Table 1).

While the specification for Criterion II was evidence-based (8, 9), its wording appears to be too restrictive and thus less useful for differentiating between trials that did not use adequate allocation concealment and trials that did so but failed to report this in full detail required by the appraisal criterion. This raises the question of whether the criterion may not be revised to be less restrictive and thus become more useful for trial appraisal without losing its empirical basis.

A simple test is presented by Hicks et al. (10) to establish whether meta-analysis results are affected by selection bias resulting from inadequate allocation concealment. The method contains the calculation of the *t*-statistic for the difference in baseline variables between treatment arms per trial; the conduct of fixed-effects meta-analysis per baseline variable is followed by the step-wise removal of trials from the meta-analysis in line with the largest *t*-statistic until heterogeneity reaches *I*^2^ = 0 and repetition of the outcome meta-analysis with trials that contributed to the heterogeneity excluded (10).

The test is based on the premise that a lack of adequate allocation concealment may lead to a biased allocation of patients to treatment groups in clinical trials. Such biased allocation will lead to imbalances in baseline variables (such as patients' age) between the groups and thus elevate the in-between-trial heterogeneity (*I*^2^ > 0) in a fixed-effects meta-analysis of baseline variables. If, after the removal of the trials (that caused the elevated heterogeneity in the baseline meta-analysis) from the outcomes meta-analysis, the result differs in effect direction and/or magnitude from that of the original (outcomes) meta-analysis result, then the latter has been biased and the test result is considered to be positive.

This study aimed to revise Criterion II of the CQS-2 with the objectives as follows:

(i) To empirically test whether meta-analyses of trials with inadequate allocation concealment generate positive test results.
(ii) To logically deduce from meta-analyses with positive test results criteria for adequate allocation concealment.
(iii) To revise Criterion II of the CQS-2 accordingly.

## 2. Methods

This study is a partial update of findings from our previous systematic review of meta-epidemiological studies concerning the CQS appraisal criterion for allocation concealment (5). In this review, we established evidence from two meta-epidemiological studies (8, 9) as a basis for formulating Criterion II of the CQS-2.

In this present study, we investigate the meta-analyses on which the two meta-epidemiological studies (8, 9) were based, for more precise data and wording, to revise the CQS-2/Criterion II into a more practical version. The methodology of this study was pre-specified in a protocol, which was made available online before the start of the study (11).

### 2.1. Literature re-review

The authors re-reviewed the two meta-epidemiological studies (8, 9) that formed the evidence base for Criterion II of the CQS-2 (5). Both studies were scanned for included meta-analyses. Meta-analyses that were found to provide evidence that trials with inadequate/unknown allocation concealment have exaggerated the true treatment effect were retrieved in full copy. The cutoff for meta-analysis selection was set as a point effect estimate of the treatment effect size (ES) > 0. The reason was that a point effect estimate =/ < 0 may have been due to either the possibility that allocation concealment has been adequately applied, but this has not been adequately reported, or that inadequate allocation concealment did not translate into a biased exaggeration of the treatment effect. In either case, a lack of individual trial baseline imbalances would have caused the test to be negative and thus rendered these meta-analyses unsuitable for testing.

The minimum number of trials required to be included in a meta-analysis was set to four, due to the following considerations:

(i) Although the test by Hicks et al. (10) requires the step-wise exclusion of trials, and thus large meta-analyses are of advantage, small meta-analyses are very common (12). Hence, a too-high minimum number of trials as a selection criterion may have excluded a too-large amount of data.
(ii) The number of trials may not be < 4 per meta-analysis because of the risk that the point estimate for heterogeneity (*I*^2^) may be too imprecise and biased when too few studies are included in a meta-analysis (12).

All full copies of the meta-analysis reports were reviewed in line with the following selection criteria:

(i) A minimum number of four trials with inadequate or unknown allocation concealment is included per forest plot.
(ii) Computable datasets concerning the effect estimates for at least one test and control group per trial reported for dichotomous data [number of events (*n*), total number of subjects (*N*)] and continuous data [total number of subjects (*N*), mean values with standard deviation (SD), or standard error (SE)].
(iii) All trials report patients' age as the baseline variable (with *N*, mean, and SD values) for test and control group(s).

All suitable trial reports that complied with criteria (i)–(iii) were retrieved in full copy.

One reviewer (SM) conducted the selection of the meta-analysis reports, and a second reviewer (VY) double-checked whether the selected reports complied with all listed criteria. Any discrepancies were resolved by discussion and consensus.

### 2.2. Data extraction

Computable datasets concerning the effect estimates per trial were extracted from the selected meta-analysis reports; datasets concerning the baseline variable “age” were extracted from the full copies of their trial reports. All data were entered into an MS Excel file. One reviewer (SM) extracted all information. A second reviewer (VY) double-checked the extracted data and corrected possible entry errors.

### 2.3. Statistical analysis

All selected meta-analyses were tested for baseline heterogeneity (*I*^2^ in %) concerning the baseline variable “age”, following the test method presented by Hicks et al. (10).

All meta-analyses were conducted using Review Manager 5.0.24 software. Baseline variable meta-analysis was conducted using the inverse variance method with a fixed effect model, while outcome meta-analysis of dichotomous data was conducted using the Mantel-Haenszel method with a random effect model.

### 2.4. Deductive inference from the test results

We started our inference with the proposition that if the test by Hicks et al. (10) yielded a positive result (¬A), then allocation concealment has not been adequate (¬B). This means that if ¬A proves to be true, then ¬B is also true. Accordingly, the equivalent proposition was made that if the test yields a negative result (A), then allocation concealment has been adequate (B). However, when trial appraisal establishes that allocation concealment was not adequate (¬B), then the test result will be positive (¬A). Furthermore, if the test is positive (¬A), then a fixed-effects meta-analysis of baseline variables will show in-between-trial heterogeneity (*I*^2^ > 0) (¬C), an imbalance between baseline variables between groups (¬D) and, therefore, a biased allocation of patients into the treatment groups (¬E). If the allocation is biased (¬E), then clinical trial results are not valid (¬F). Therefore, the concluding proposition was made that if allocation concealment was not adequate (¬B), then clinical trial results were not valid (¬F). From this follows that non-compliance of trial characteristics with the criterion for adequate allocation concealment falsifies clinical trial results. This line of deductive reasoning was based on the rules of propositional logic: modus pones, modus tollens, and the law of implication reversal (Additional file) (13). Based on this, the criterion was established for adequate allocation concealment, against which compliance and thus trial validity can be tested and falsified during trial appraisal.

### 2.5. Revision of the CQS-2/Criterion II

We extracted the appraisal criteria verbatim for “adequate allocation concealment” that were applied in the meta-analyses with positive test results and also, if available, the descriptions of how allocation concealment was conducted in the trials of the meta-analyses with adequate allocation concealment and recorded them in a verbatim table.

The extracted verbatim text was analyzed for the main keywords used, as well as their intent of meaning. The CQS-2/Criterion II was reformulated in accordance with these established records. The revised appraisal tool was specified as CQS-2B.

## 3. Results

**Figure 1:**
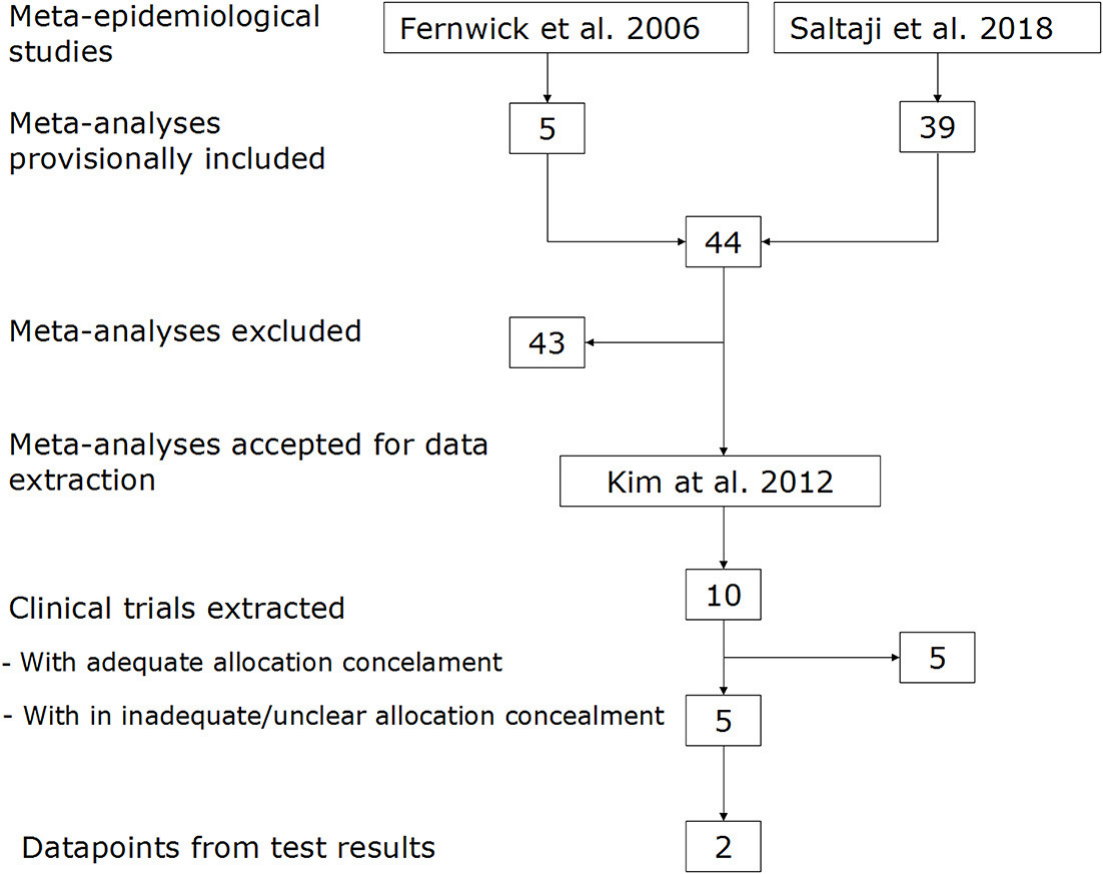
Flow diagram of identification, screening, and inclusion of meta-analyses and datapoints.

From the two meta-epidemiological studies (8, 9), a total of 44 meta-analyses were included (Figure 1). From these, 43 were excluded for the following reasons: point effect estimate of the treatment effect size (ES) =/ < 0 (18); too few trials (< 4) included in the analysis (17); not all trials traceable (2); no computable data reported (3); no allocation concealment appraisal reported (1); and duplication (2). The references of all excluded meta-analyses are listed in the Additional file.

One meta-analysis by Kim et al. could be accepted for data extraction (14). From this report, two forest plots were selected for data extraction that included five and four trials with inadequate/unclear allocation concealment, which were included in analyses 1 and 2, respectively. In addition, a total of five trials with adequate allocation concealment were identified (Supplementary material).

The results of the conducted meta-analyses of the baseline variable “age” with the calculated *t*-statistics for differences between the randomized groups are presented in Table 2, and the results of the subsequent outcome meta-analyses are in Table 3.

**Table 2:** Trials included in the meta-analysis of baseline age by *t*-statistic for the difference in age between randomized groups.

**Trial**	**MD**	***t*-statistic (Absolute value)**	Heterogeneity *I*^2^ (%)^*^
**Analysis 1**			**21% total**
Lopez et al. (15)	1.00	2.13	0.0
Offenbacher et al. (16)	1.10	0.87	0.0
Macones et al. (17)	−0.30	0.75	0.0
Tarannum and Faizuddin (18)	0.10	0.20	0.0
Sadatmansouri et al. (19)	0.70	0.05	0.0
**Analysis 2**			**35% total**
Lopez et al. (15)	1.00	2.13	0.0
Macones et al. (17)	−0.30	0.75	0.0
Tarannum and Faizuddin (18)	0.10	0.20	0.0
Sadatmansouri et al. (19)	0.70	0.05	0.0

^*^Heterogeneity was observed when the trial with a higher *t*-statistic was removed. MD, mean difference.

**Table 3:** Results of the outcome meta-analyses.

**Analysis Nr**.	**Meta-analysis**	**No. of trials**	**RR (95% CI)**	***p*-value**	**Heterogeneity *I*^2^ (%)**
1	Original	11	0.81 (0.64–1.02)	0.07	59.0
	Replicated^a^	5	0.66 (0.45–0.98)	0.04	49.0
	After identified trials removed	4	0.72 (0.51–1.02)	0.07	43.0
2	Original	7	0.97 (0.75–1.24)	0.79	37.0
	Replicated^a^	4	0.54 (0.19–1.55)	0.25	84.0
	After identified trials removed	3	0.68 (0.22–2.11)	0.50	88.0

^a^Replicated with trials with inadequate/unclear allocation concealment, only. RR, relative risk; CI, confidence interval.

The replicated results for analyses 1 and 2 were RR 0.61 (95% CI: 0.45–0.98; *p* = 0.04) and RR 0.54 (95% CI: 0.19–1.55; *p* = 0.25), respectively. The results after trial removal were RR 0.72 (95% CI: 0.51–1.02; *p* = 0.07) and RR 0.68 (95% CI: 0.22–2.11; *p* = 0.50), respectively. According to Hicks et al. (10), the results of both analyses show a change in effect magnitude and direction after identified trials had been removed, thus indicating a positive test result. Such positive results provide confirmation for an imbalance in the distribution of the baseline variable “age” between randomized groups, due to biased patient allocation.

The positive test results empirically corroborated the assumption that meta-analysis results from clinical trials, which did not comply with the criteria for adequate allocation concealment, are likely to be biased. Accordingly, criteria for adequate allocation concealment extracted verbatim from the accepted meta-analysis (14) and its individual trials (20–24) (Table 4).

**Table 4:** Verbatim table of extracted text quotes.

**Meta-analysis:**	**Kim et al. (14)**				
Applied criterion for “adequate allocation concealment”	• “Methodologic quality assessment risk of bias tool was devised based on Cochrane Handbook” (25) (Page 1,510/Left Column/Paragraph 3/Line 4). • Ref. (25): Higgins JPT, Green S. *Cochrane Handbook for Systematic Reviews of Interventions v.5.0.2*. The Cochrane Collaboration (2009). Available online at: http://www.cochrane-handbook.org (accessed May 1, 2011). • “Participants and investigators enrolling participants could not foresee assignment because one of the following, or an equivalent method, was used to conceal allocation: ° Central allocation (including telephone, web-based, and pharmacy-controlled randomization); ° Sequentially numbered drug containers of identical appearance; ° Sequentially numbered, opaque, sealed envelopes.”
**Trials**	**Radnai et al**. **(****20****)**	**Jeffcoat et al**. **(****21****)**	**Offenbacher et al**. **(****22****)**	**Michalowicz et al**. **(****23****)**	**Newnham et al**. **(****24****)**
Description of how allocation concealment was conducted	No information in the published trial report.	Page 1,216/Left Column/Paragraph 3/Line (1–5): “A UAB research pharmacist, who provided a double packet with coding information for each patient, generated the randomization code. The clinicians delivering periodontal care had no role in determining the outcome of the study.”	No information in the published trial report.	Page 1,886/Right Column/Paragraph 3/Line (1–4): “Randomization, stratified by the center with the use of permuted randomized blocks of 2 and 4, was made by a telephone call to the coordinating center.”	No information in the published trial report.

While three of the trials did not provide relevant information (20, 22, 24), the main keywords of the meta-analysis and two trials (14, 21, 23) indicate that “central allocation” (in some form, whether it be by telephone, web-based, or pharmacy-controlled and translated by sequentially numbered, identical containers or sequentially numbered, identical, opaque, sealed envelopes, or other forms) provides adequate allocation concealment.

The intent of the meaning of the keyword “central allocation” was taken as being of “any assurance in the text that the patient allocation according to the random sequence was applied by an independent agent or agency, not otherwise involved in the trial”. Accordingly, the Criterion II of the CQS-2 was revised (Table 5).

**Table 5:** CQS-2B appraisal criteria.

Criterion I	“Randomization” for allocation to treatment groups is in some form reported in the text
**Criterion II**	**Any assurance that the patient allocation to treatment groups according to the random sequence was applied by an independent agent or agency, not otherwise involved in the trial, is in some form reported in the text**
Criterion III	Double-blinding or the blinding of at least two out of the three groups: trial participants, trial personnel, and trial outcome assessors in some form reported in the text
Criterion IV	The sample size of any particular treatment group reported in the trial is not < *N* = 100

Bold indicates the revised appraisal criterion.

## 4. Discussion

This study aimed to revise Criterion II of the CQS-2 trial appraisal tool. To establish an empirical basis for this revision, we first tested whether meta-analyses of trials with inadequate allocation concealment generate positive test results. One meta-analysis with positive test results could be identified from which we logically deduced the criterion for adequate allocation concealment and, in accordance with its verbatim text, subsequently formulated the new Criterion II.

During our review, we excluded 43 out of 44 meta-analyses as unsuitable for testing. Therefore, the results of the single accepted meta-analysis (14) provide only a limited empirical basis for revising CQS-2/Criterion II. Furthermore, it was surprising that three of the five trial reports (20, 22, 24) labeled by the authors of the meta-analysis (14) as adequate allocation concealment did not contain information in that regard. We assumed that Kim et al. obtained this information by contacting the trial authors after these trial reports had been published. However, for our study, we could thus rely, in addition to the meta-analysis text (14), on two trial reports (21, 23) only.

Despite these limitations, our study could establish the revised Criterion II (CQS-2B) on a more precise empirical basis than for the original CQS version (CQS-2). While the latter relied on the data and wording of meta-epidemiological evidence (5, 8), the former is based on the data and wording of one meta-analysis (14) from which the meta-epidemiological evidence (8) was established. Further precision might be achieved if individual randomized trials themselves could be tested for potential selection bias instead of meta-analyses of such trials. However, to our knowledge, only two test methods are currently available for this purpose, namely baseline testing within a trial and the Berger-Exner test. While baseline testing in individual trials may give some indication for allocation problems (25), it may generate misleading findings (10, 26). The highly accurate Berger-Exner test (27) relies on raw data that are mostly accessible to the trial's authors only and thus can be conducted (and its results reported) by only the trial authors themselves. In contrast, the bias test presented by Hicks et al. (10) enables application by reviewers to empirically ascertain whether the results of meta-analyses of trials are biased. Since such meta-analyses also include the appraisal of bias risk, for example, whether allocation concealment in trials was adequate or not, the wording of their appraisal criteria is more precise in relation to empirical bias test findings than the wording from meta-epidemiological studies that pooled several meta-analyses with differences in the wording of each of their appraisal criteria.

It has been suggested that basing trial appraisal criteria on empirical results from meta-epidemiological studies may be futile because most of these studies can control only incompletely for confounding and, subsequently, their results cannot be ascribed to the lack or incomplete reporting of a particular trial's characteristics. Instead, the reliance on theoretical justification was suggested (28).

However, the sole theoretical justification lacks information on whether the theory corresponds with empirical facts. When a theory is compared with empirical facts, such comparison constitutes a test. If the test outcome is negative, then empirical facts are shown to contradict the theory, which in turn is then considered falsified. Such falsification is sufficient not to accept the theory. If the test result is positive, the hypothesis is considered corroborated. This does not mean that the theory is true but only indicates that it has passed the test for now and there is thus no current reason to reject it. As long as the theory remains corroborated, it can explain reality well and does not conflict with empirical facts for the time being.

In our study, we could establish from the limited available data, so far, that meta-analysis results of pooled trials with inadequate/unclear allocation concealment correspond with positive test results in terms of in-between-trial heterogeneity (*I*^2^ > 0) due to imbalances in the baseline variable “age” between randomized groups. From these results, we logically deduced criteria for adequate allocation concealment, relating to the need for central allocation of patients according to the random allocation sequence.

The findings of our study are in line with past and current guidelines for assessing the risk of bias in randomized trials by the Cochrane Collaboration. The Cochrane Reviewers' Handbook version 4.2.1 in 2003 already stated that “the ideal is for the process to be impervious to any influence by the individuals making the allocation. This will be most securely achieved if an assignment schedule generated using true randomization is administered by someone who is not responsible for recruiting subjects, such as someone based in a central trial office or pharmacy.” (29). In 2006, the handbook version 4.2.6 maintains that “centralized (e.g., allocation by a central office unaware of subject characteristics) or pharmacy-controlled randomization” is one of the approaches that can be used to ensure adequate concealment (30). From 2017 until now (2022), subsequent handbook versions have maintained that “central randomization by a third party is perhaps the most desirable” (31–37).

## 5. Recommendations for future research

The CQS is still in development. Since only limited data for the revision of Criterion II were found, it is recommended that subsequent investigations expand the search for further empirical evidence to meta-analyses beyond that of the current two meta-epidemiological studies (8, 9).

In addition, prospective, controlled clinical therapy trials from systematic reviews that have applied the 2nd version of Cochrane's RoB tool may be re-appraised using the new CQS-2B version to establish whether the direction and magnitude of any pooled effect estimates remain the same. Such investigation may statistically compare the different pooled effect estimates using the Wald test by testing the null hypothesis that both are, at a significance level of 5%, not significantly different. Furthermore, clinical conclusions for all measured outcomes may be qualitatively compared by the use of a comparison table.

Based on the results of these further investigations, the CQS-2B may be piloted as part of the regular, systematic review methodology for the appraisal of prospective, controlled clinical therapy trials.

## 6. Conclusion

Based on this study's result, the Criterion II of the CQS-2 trial appraisal tool was revised as follows: “Any assurance that the patient allocation to treatment groups according to the random sequence was applied by an independent agent or agency, not otherwise involved in the trial, is in some form reported in the text”. The revised appraisal tool was specified as version CQS-2B.

## Data availability statement

The original contributions presented in the study are included in the article/Supplementary material, further inquiries can be directed to the corresponding author.

## Author contributions

SM contributed to the conception and design of the study, performed the statistical analysis, and wrote the first draft of the manuscript. VY commented on and improved the manuscript. Both authors read and approved the final version of the manuscript.
